# Beneficial Role of Replacing Dietary Saturated Fatty Acids with Polyunsaturated Fatty Acids in the Prevention of Sarcopenia: Findings from the NU-AGE Cohort

**DOI:** 10.3390/nu12103079

**Published:** 2020-10-09

**Authors:** Diego Montiel-Rojas, Aurelia Santoro, Andreas Nilsson, Claudio Franceschi, Miriam Capri, Alberto Bazzocchi, Giuseppe Battista, Lisette C. P. G. M. de Groot, Edith J. M. Feskens, Agnes A. M. Berendsen, Agata Bialecka-Debek, Olga Surala, Barbara Pietruszka, Susan Fairweather-Tait, Amy Jennings, Frederic Capel, Fawzi Kadi

**Affiliations:** 1 School of Health Sciences, Örebro University, 702 81 Örebro, Sweden; diego.montiel@oru.se (D.M.-R.); fawzi.kadi@oru.se (F.K.); 2 Department of Experimental, Diagnostic and Specialty Medicine, Alma Mater Studiorum, University of Bologna, 40138 Bologna, Italy; aurelia.santoro@unibo.it (A.S.); claudio.franceschi@unibo.it (C.F.); miriam.capri@unibo.it (M.C.); g.battista@unibo.it (G.B.); 3 Alma Mater Research Institute on Global Challenges and Climate Change (Alma Climate), University of Bologna, 40126 Bologna, Italy; 4 Department of Applied Mathematics, Institute of Information Technology, Mathematics and Mechanics (ITMM), Lobachevsky State University of Nizhny Novgorod-National Research University (UNN), Nizhny Novgorod 603950, Russia; 5 Diagnostic and Interventional Radiology, IRCCS Istituto Ortopedico Rizzoli, 40136 Bologna, Italy; alberto.bazzocchi@ior.it; 6 Department of Human Nutrition and Health, Wageningen University, 6708WE Wageningen, The Netherlands; lisette.degroot@wur.nl (L.C.P.G.M.d.G.); edith.feskens@wur.nl (E.J.M.F.); agnes.berendsen@wur.nl (A.A.M.B.); 7 Department of Human Nutrition, Warsaw University of Life Sciences-SGGW, 02-776 Warsaw, Poland; agata_bialecka_debek@sggw.edu.pl (A.B.-D.); olga.surala@insp.waw.pl (O.S.); barbara_pietruszka@sggw.edu.pl (B.P.); 8 Norwich Medical School, University of East Anglia, Norwich NR4 7TJ, UK; S.Fairweather-Tait@uea.ac.uk (S.F.-T.); Amy.Jennings@uea.ac.uk (A.J.); 9 Unité de Nutrition Humaine (UNH), Institut National de Recherche pour L’agriculture, L’alimentation et L’environnement (INRAE), Université Clermont Auvergne, CRNH Auvergne, 63000 Clermont-Ferrand, France; frederic.capel@inrae.fr

**Keywords:** ageing, muscle mass, dietary fats, macronutrients, isocaloric substitution, muscle strength, physical activity, metabolic syndrome

## Abstract

Dietary fat subtypes may play an important role in the regulation of muscle mass and function during ageing. The aim of the present study was to determine the impact of isocaloric macronutrient substitutions, including different fat subtypes, on sarcopenia risk in older men and women, while accounting for physical activity (PA) and metabolic risk. A total of 986 participants, aged 65–79 years, completed a 7-day food record and wore an accelerometer for a week. A continuous sex-specific sarcopenia risk score (SRS), including skeletal muscle mass assessed by dual-energy X-ray absorptiometry (DXA) and handgrip strength, was derived. The impact of the isocaloric replacement of saturated fatty acids (SFAs) by either mono- (MUFAs) or poly-unsaturated (PUFAs) fatty acids on SRS was determined using regression analysis based on the whole sample and stratified by adherence to a recommended protein intake (1.1 g/BW). Isocaloric reduction of SFAs for the benefit of PUFAs was associated with a lower SRS in the whole population, and in those with a protein intake below 1.1 g/BW, after accounting for age, smoking habits, metabolic disturbances, and adherence to PA guidelines. The present study highlighted the potential of promoting healthy diets with optimised fat subtype distribution in the prevention of sarcopenia in older adults.

## 1. Introduction

Population ageing is accompanied by an increased risk of sarcopenia, a chronic condition characterised by a loss of muscle mass and strength, and associated with several adverse outcomes, including physical disability and a poor quality of life [1,2]. Fortunately, the rate of muscle mass and function decline in older adults can be reduced by appropriate nutrition and physical activity behaviours, making these modifiable lifestyle behaviours important non-pharmacological approaches for the prevention of sarcopenia [3,4].

While nutritional habits have the potential of readily impacting sarcopenia risk, the complex relationship between macronutrient composition and the regulation of muscle health is not fully established. To date, a large number of studies has focused on adequate protein intake for the maintenance of muscle mass and function, where an intake of around 1.1 g/BW has been recommended for older adults [5]. In addition to proteins, it has been hypothesized that lipid intake in general, and fat subtypes in particular, may have further influence on age-related loss of muscle mass and function [6]. For example, it has been reported that a high dietary intake of saturated fatty acids (SFAs) may exacerbate the development of sarcopenia [7].

Alongside absolute intakes of single macronutrients, the isocaloric distribution of different macronutrients and their related subtypes likely influences the regulation of muscle mass and function in older adults [8,9]. Therefore, to disentangle the interactive effect of macronutrients on muscle health, isocaloric substitution models offer the opportunity to explore the effects on health outcomes of replacing one macronutrient with another, whilst keeping the remaining relative macronutrient intakes constant. This approach accounts for the fact that an isocaloric change of one macronutrient inevitably alters the relative intakes of other macronutrients. Based on isocaloric substitution models, associations with metabolic health outcomes were previously reported in relation to different macronutrient distributions [10,11,12], and the replacement of fatty acids by either carbohydrates or proteins has previously been linked to a lower cardiovascular disease incidence [13]. Currently, there are limited data on the impact of macronutrient replacement on muscle mass and function in older adults, which is unfortunate considering the demographic shift accompanied by the increased prevalence of people with a physical disability. Importantly, given the existing protein intake recommendations for older adults, the impact of macronutrient replacement on muscle mass in older adults below and above this threshold is warranted. Notably, there is a well-established relationship between physical activity (PA) of at least moderate intensity and muscle quantity and quality. Therefore, to clarify the relationships between macronutrient distribution and muscle mass in older adults, the confounding effects of PA need also to be considered.

The aim of the present study was to explore the impact of isocaloric macronutrient substitutions, including different fat subtypes, on sarcopenia risk in a cohort of older European men and women from the NU-AGE study (New dietary strategies addressing the specific needs of elderly population for an healthy ageing in Europe).

## 2. Materials and Methods 

### 2.1. Participants

The present study included 986 older men and women, aged 65–79 years, recruited within the frame of the NU-AGE project (Clinicaltrials.gov, NCT01754012) at baseline (April 2012–January 2014). A detailed description of the recruitment process and study design has been described elsewhere [14,15]. Participants fulfilling the frailty criteria [16], with a disability or overt disease at screening, were excluded. Local ethical approval was provided by the Independent Ethics Committee of the Sant’Orsola-Malpighi Hospital Bologna (Italy-03/2011/U/Sper), the National Research Ethics Committee East of England (UK-12/EE/0109), the Wageningen University Medical Ethics Committee (Netherlands-11/41 NU-AGE), and the Bioethics Committee of the Polish National Food and Nutrition Institute (Poland). Written informed consent was obtained, and the study was conducted in accordance with the standards set by the Declaration of Helsinki. 

### 2.2. Dietary Intake

Dietary intake was assessed using a food record as previously described [14,17]. Participants completed a 7-day food record and had an interview with a trained dietician/research nutritionist to review the records. Consumed foods were coded according to standardised procedures and translated into nutrients by the use of software exploiting local food composition tables [14,17,18]. Macronutrient intakes of carbohydrates, proteins, and fats, including saturated fatty acids (SFAs), monounsaturated fatty acids (MUFAs), and polyunsaturated fatty acids (PUFAs), were derived. Energy intake from macronutrients was normalised against body weight (kcal/BW).

### 2.3. Body Composition

Height and weight were measured using standardised procedures, and body composition was assessed using dual-energy X-ray absorptiometry (DXA), as described previously [19,20]. DXA scans were performed by trained personnel. The analytical program defined six corporeal regions, where total and regional fat and lean masses were derived. Skeletal muscle mass index (SMI, %) was calculated as previously described [19,20,21,22].

### 2.4. Handgrip Strength and Physical Limitations

Handgrip strength, adjusted by body weight, was determined with a Jamar handheld dynamometer (Patterson Medical, Warrenville, IL, United States) using standardised procedures. Participants were also classified as having or not having physical function (PF) limitations by the 10-item PF subscale of the 36-item Short Form Health Survey (SF-36) [23], as described elsewhere [24].

### 2.5. Sarcopenia Risk Score

A continuous clustered sarcopenia risk score (SRS) was created based on SMI and handgrip strength, according to the most recent operational definition of sarcopenia [1]. First, sex-specific standardised values of SMI and handgrip strength were calculated and averaged into composite z-scores, where higher scores indicated a higher sarcopenia risk.

### 2.6. Adherence to Physical Activity Guidelines

Time spent in moderate-to-vigorous PA (MVPA) was assessed using a waist-worn Actigraph accelerometer (GT3x activity monitor, Actigraph, Pensacola, FL, USA) for a week. As previously described [25], the monitor had to be worn for at least 4 days, with at least 10 h per day for inclusion, and non-wear time was defined as 60 min of continuous zero counts. The count cut-point used to determine MVPA time was based on previous work [26]. Adherence to PA guidelines (≥150 weekly minutes of MVPA) was approximated to a daily average of ≥22 min in MVPA.

### 2.7. Assessment of Metabolic Risk

Participants were classified as with or without metabolic syndrome (MetS) based on sex-specific definitions set by the International Diabetes Federation [27]. In brief, waist circumference (WC) was determined at the midpoint between the iliac crest and lower costal margin to the nearest 0.1 cm. Systolic and diastolic blood pressures were assessed using an automated electronic blood pressure monitor as previously described [20]. All biochemical analyses, including blood glucose and blood lipids (triglycerides, high-density lipoprotein (HDL) and low-density lipoprotein (LDL) cholesterol), were performed in one centre based on standard methodologies.

### 2.8. Statistical Analysis

The data are presented as arithmetic mean and standard deviation unless otherwise indicated. Differences between male and female participants were determined by either independent sample *t*-tests or chi-square tests. Partial correlation was used to investigate the relationship between macronutrient intake and SRS, adjusting for total energy intake. Linear regression modelling was used to assess the hypothetical change in SRS by an isocaloric replacement of macronutrients. First, the effects of altering macronutrient distribution were achieved by including the total energy intake together with energy intakes from two macronutrients, while leaving out the third. Second, the effects of reducing intakes of SFAs were achieved by including energy from MUFAs and PUFAs, while keeping the remaining energy-providing nutrients unchanged. All models were adjusted by age, recruiting centre, smoking habit, fibre intake (g/day), the prevalence of MetS, and adherence to PA guidelines. Given the likely influence of protein intake on SRS, isocaloric substitution modelling of SFAs by other fat subtypes (MUFAs and PUFAs) was further stratified based on a protein intake of 1.1 g/BW. All assumptions behind regression analyses including normality, linearity, homoscedasticity, and multicollinearity were checked. Based on our sample size, a priori power calculation revealed that small-to-moderate effect sizes were detectable in SRS with a power of >80% and alpha set to 0.05. All analyses were conducted using SPSS, version 26.

## 3. Results

The general characteristics of the study population are presented in Table 1. Male participants had significantly higher handgrip strength and SMI compared to females (Table 1). Further, a significantly higher proportion of males adhered to PA guidelines, with a lower proportion reporting a physical function limitation compared to females (Table 1). There were no significant sex differences in body mass index (BMI), or MetS prevalence (Table 1).

Total energy intake in the whole population was 1809 ± 419 kcal/day (Table 2), with an average of 49% of energy derived (E%) from carbohydrates, 17 E% from protein, and 31 E% from fat (12 E%, 13 E%, and 6 E% for SFAs, MUFAs, and PUFAs, respectively). After adjustment by body weight, total energy and carbohydrate intake were significantly higher in the male participants (*p* < 0.05), whereas no corresponding differences were observed for protein, total fat, or its subtypes. Approximately two-thirds (66%) of all participants had a protein intake less than 1.1 g/BW, and 75% had an energy intake of SFAs above 10 E%.

Partial correlation analysis showed inverse associations between energy intake from all macronutrients and SRS (*p* < 0.05), after controlling for total energy intake.

Isocaloric substitution models showed that replacing total fat by a given amount of either protein or carbohydrates was significantly associated with a reduced risk of sarcopenia (Table 3). However, an isocaloric substitution of total carbohydrates by protein was not associated with SRS (β-Coeff. −0.037, 95% CI (−0.108 to 0.034), *p* = 0.305).

Further analysis revealed a reduced SRS when replacing SFAs by PUFAs in isocaloric models, whereas no corresponding effect was evident when substituting SFAs by MUFAs (Table 4). Additionally, we sought to investigate whether meeting the recommended daily amount of 1.1 g/BW of protein may alter the associations between SRS and the distribution of fat subtypes. Interestingly, an isocaloric substitution of SFAs by PUFAs resulted in lower SRS only in participants with a protein intake below 1.1 g/BW (Table 4).

## 4. Discussion

The present study highlighted the beneficial impact of replacing SFAs with PUFAs on the risk of sarcopenia in older European adults. This is of particular importance in older men and women with a protein intake below the recommended amount of 1.1 g/BW. Furthermore, the impact of dietary fat quality on sarcopenia risk was evident regardless of adherence to PA guidelines and metabolic risk status, which suggests that dietary fat quality plays a pivotal role in the prevention of sarcopenia in older adults. 

Our study revealed that the replacement of total fat intake at the expense of other macronutrients is related to a lower sarcopenia risk. Recent reports have shown that high-fat diets are associated with an increased sarcopenia risk in ageing populations [28,29], suggesting that the distribution of fat subtypes may explain the increased sarcopenia risk. To test this hypothesis, we further analysed the impact of the replacement of SFAs by either MUFAs or PUFAs on sarcopenic risk using isocaloric modelling. A major finding was that the replacement of SFAs with PUFAs, but not MUFAs, was related to a significant reduction in sarcopenia risk, which supports that the type of fat, as well as the relative distribution, should be emphasized in dietary strategies against sarcopenia progression. In accordance with our findings, previous studies investigating the influence of fat subtypes on single components of sarcopenia risk (e.g., muscle mass and function) showed that SFA intake is linked to a higher risk of functional impairment [30] and lower physical function [31]. Furthermore, positive associations of higher intakes of PUFA-rich food and muscle mass and function have been shown in a population of ≥60-year-old men and women [32,33]. At the cellular level, several molecular pathways leading to muscle wasting may explain the detrimental action of SFAs. For example, exposure of muscle cells to SFAs induced reduction in cell size and suppression in insulin signalling, together with an increased expression of pro-atrophic genes [34,35]. Another pathway by which SFAs may modulate muscle mass is their ability to downregulate the activity of key nutrient transporters, which can impair amino acid uptake and thus facilitate muscle mass loss [36]. In parallel, PUFAs may promote muscle hypertrophy through enhanced activation of the mammalian target of rapamycin (mTOR) growth pathway and downregulation of the pro-inflammatory mediator IL-1β [37,38]. To date, the effects of MUFAs on muscle mass and function are inconclusive. For instance, positive, negative, and no relationships were reported between intakes of MUFAs and indices of muscle health [6,30,39]. In light of our results and previous literature, further investigations including experimental designs are warranted.

Given the well-established role of adequate protein intake on the maintenance of muscle mass, we further investigated whether the impact of fat subtype distribution on sarcopenia risk is moderated by adherence to a recommended protein intake of 1.1 g/BW. Interestingly, while the detrimental impact of SFAs was suppressed in older men and women meeting the recommended intake, it was still observed at protein intakes below this quantity. In our sample of older European adults, approximately two-thirds had a protein intake below 1.1 g/BW, which is in line with a recent report on the prevalence of older adults with inadequate protein intake [40]. Thus, given that a substantial proportion of older adults do not consume recommended protein intakes, our findings strengthen the need to consider diets with favourable fat subtype distribution in order to reduce sarcopenia risk.

Together with healthy dietary patterns, PA is regarded as a key lifestyle factor that can readily have an impact on muscle mass and function, where 150 weekly minutes of MVPA is the guideline endorsed by major health organizations [41]. Therefore, adherence to this PA guideline should be considered when exploring diet-related health effects. The present study demonstrated that the detrimental impact of SFAs on sarcopenia risk is evident regardless of adherence to the PA guidelines. This finding has important implications in terms of public health strategies, where efforts need to include optimization of dietary fat intakes alongside adequate protein intake and health-enhancing PA behaviours.

The population sample of the present study comprised older men and women of diverse geographical and cultural origins and with different metabolic health status (with or without MetS). Interestingly, the beneficial impact of replacing SFAs with PUFAs on sarcopenia risk occurred regardless of these study sample variations, which strengthens the need to optimize dietary fat intake irrespective of culturally related dietary differences or stages of metabolic disease progression.

The main findings of the present study were strengthened by the use of objective assessment of PA together with a food record-based assessment of macronutrient intakes. Sarcopenia risk was assessed by incorporating single elements (SMI and handgrip strength) into one composite score according to recent operational definitions of sarcopenia [1], which is likely to better capture different stages of sarcopenia progression than separate single parameters of muscle mass and strength. The present study was not without limitations. The cross-sectional design prevented conclusions about causality. Further experimental work is warranted to confirm the findings of this study. While food records are regarded as valid for determining energy intakes, over- and under-reporting likely occur. The data on macronutrient intakes were in line with data from previous reports in older adults [3,28,42], and general over- or under-reporting in this population would not affect the validity of the study conclusions. Although several covariates were included in the main analyses, residual confounding from other variables cannot be ruled out.

## 5. Conclusions

The present study suggested a beneficial impact of replacing SFAs with PUFAs, but not MUFAs, on the sarcopenia risk score in non-frail older men and women, especially in those with a protein intake below the current recommendation. Efforts to promote healthy diets with optimised fat subtype distribution should be emphasised regardless of adherence to PA guidelines.

## Figures and Tables

**Table 1:** General characteristics of the study population.

	Total	Male	Female
n	986	417	569
**Basic Characteristics**			
Age (years)	71 ± 4	71 ± 4	71 ± 4
Weight (kg)	74.7 ± 13.4	82.4 ± 12	69.1 ± 11.3 *
Height (cm)	165 ± 9	173 ± 6	160 ± 7 *
BMI (kg/m^2^)	27.0 ± 4.0	27.2 ± 3.7	26.8 ± 4.2
SMI (%)	27.0 ± 4.3	30.6 ± 3.2	24.4 ± 2.8 *
Full Education (years)	13 ± 4	13 ± 4	12 ± 3 *
Smoking (% never)	51.3	37.6	61.3 *
Medication (% yes)	77.6	77.5	77.7
PA Guidelines (% yes)	54.1	63.8	46.9 *
**Physical Function**			
Handgrip Strength (kg/BW)	0.42 ± 0.11	0.49 ± 0.09	0.38 ± 0.09 *
Physical Limitation (% yes)	33.8	22.1	42.4 *
**Metabolic Risk Factors**			
MetS (% yes)	41.7	44.6	39.5
Waist Circumference (cm)	92.4 ± 11.7	98.0 ± 10.6	88.3 ± 10.8 *
SBP (mmHg)	140 ± 20	141 ± 18	139 ± 21
DBP (mmHg)	75 ± 11	77 ± 10	74 ± 11 *
Glucose (mmol/L)	5.57 ± 0.83	5.75 ± 0.94	5.43 ± 0.71 *
Triglycerides (mmol/L)	1.07 ± 0.47	1.08 ± 0.49	1.06 ± 0.45
HDL-cholesterol (mmol/L)	1.53 ± 0.47	1.32 ± 0.36	1.71 ± 0.47 *
LDL-cholesterol (mmol/L)	3.31 ± 0.96	3.13 ± 0.93	3.47 ± 0.98 *

Continuous data are expressed as mean ± SD, or are otherwise indicated. BMI: body mass index; SMI: skeletal muscle mass index; BW: body weight; PA: physical activity; MetS: metabolic syndrome; DBP: diastolic blood pressure; SBP: systolic blood pressure; HDL: high-density lipoprotein; LDL, low-density lipoprotein. * *p* < 0.05 vs. male.

**Table 2:** Daily energy and macronutrient intake of the study population.

	Total	Male	Female
n	986	417	569
**Nutritional Intake**			
Total Energy (kcal)	1809 ± 419	2037 ± 433	1642 ± 319 *
Carbohydrates (g)	221.1 ± 61.5	250.0 ± 66.6	200.0 ± 47.6 *
Fat (g)	62.7 ± 19.1	69.4 ± 20.4	57.8 ± 16.4 *
SFAs (g)	24.9 ± 9.4	27.1 ± 10.0	23.3 ± 8.7 *
MUFAs (g)	26.1 ± 8.4	29.5 ± 9.2	23.7 ± 6.9 *
PUFAs (g)	11.7 ± 5.1	12.8 ± 5.4	10.8 ± 4.7 *
Protein (g)	74.5 ± 17.7	82.1 ± 19.2	68.9 ± 14.2 *

Continuous data are expressed as mean ± SD. SFAs: saturated fatty acids; MUFAs: monounsaturated fatty acids; PUFAs: polyunsaturated fatty acids. * *p* < 0.05 vs. male.

**Table 3:** Effect of isocaloric substitution of fat with either protein or carbohydrates on sarcopenia risk score in older European adults.

	Sarcopenia Risk Score		
Model	β-Coeff.	95% CI	*p*-Value
Protein	−0.077	−0.152 to −0.003	0.042
Carbohydrates	−0.040	−0.07 to −0.008	0.015

CI: confidence interval. Substitution model contains total energy intake (Kcal/BW), protein intake (Kcal/BW), carbohydrates intake (Kcal/BW), alcohol intake (Kcal/BW), and fibre intake (g/day). Models were additionally adjusted for age, recruiting centre, smoking habits, meeting the recommendations of physical activity (yes/no), and prevalence of metabolic syndrome (yes/no). Estimates were interpreted as the association of the SRS with a 1 Kcal/BW increase of the substituent macronutrients (protein or carbohydrates) to the detriment of fat, while keeping the remaining constant. Analysed based on n = 986.

**Table 4:** Effect of the isocaloric substitution of saturated fatty acids by unsaturated fatty acids on sarcopenia risk score in the whole population of older European adults and stratified by meeting the recommendation of 1.1 g/BW of protein intake.

	Sarcopenia Risk Score		
Model	β-Coeff.	95% CI	*p*-Value
**Whole Population**			
MUFAs	−0.012	−0.121 to 0.097	0.829
PUFAs	−0.152	−0.253 to −0.051	0.003
**Protein Intake < 1.1 kg/BW**			
MUFAs	−0.012	−0.168 to 0.144	0.879
PUFAs	−0.162	−0.303 to −0.020	0.025
**Protein Intake ≥ 1.1 kg/BW**			
MUFAs	−0.067	−0.227 to 0.094	0.417
PUFAs	−0.093	−0.241 to 0.056	0.221

CI: confidence interval; MUFAs: monounsaturated fatty acids; PUFAs: polyunsaturated fatty acids; BW: body weight. Substitution model contains total energy intake (Kcal/BW), protein intake (Kcal/BW), carbohydrates intake (Kcal/BW), MUFAs intake (Kcal/BW), PUFAs intake (Kcal/BW), alcohol intake (Kcal/BW), and fibre intake (g/day). Models were additionally adjusted for age, recruiting centre, smoking habits, meeting the recommendations of physical activity (yes/no), and prevalence of metabolic syndrome (yes/no). Estimates were interpreted as the association of the SRS with a 1 Kcal/BW increase of the substituent macronutrients (MUFAs or PUFAs) to the detriment of SFAs while keeping the remaining constant. Analysed based on n = 986.
